# An International Survey of Deep Brain Stimulation Utilization in Asia and Oceania: The DBS Think Tank East

**DOI:** 10.3389/fnhum.2020.00162

**Published:** 2020-07-06

**Authors:** Chencheng Zhang, Adolfo Ramirez-Zamora, Fangang Meng, Zhengyu Lin, Yijie Lai, Dianyou Li, Jinwoo Chang, Takashi Morishita, Tooru Inoue, Shinsuke Fujioka, Genko Oyama, Terry Coyne, Valerie Voon, Paresh K. Doshi, Yiwen Wu, Jun Liu, Bhavana Patel, Leonardo Almeida, Aparna A. Wagle Shukla, Wei Hu, Kelly Foote, Jianguo Zhang, Bomin Sun, Michael S. Okun

**Affiliations:** ^1^Department of Functional Neurosurgery, Ruijin Hospital, Shanghai Jiao Tong University School of Medicine, Shanghai, China; ^2^Norman Fixel Institute of Neurological Diseases, Program for Movement Disorders and Neurorestoration, Department of Neurology, University of Florida College of Medicine, Gainesville, FL, United States; ^3^Department of Neurosurgery, Tiantan Hospital, Capital Medical University, Beijing, China; ^4^Department of Neurosurgery, Yonsei University College of Medicine, Seoul, South Korea; ^5^Department of Neurosurgery, Fukuoka University, Fukuoka, Japan; ^6^Department of Neurology, Fukuoka University, Fukuoka, Japan; ^7^Department of Neurology, Juntendo University Graduate School of Medicine, Tokyo, Japan; ^8^Queensland Brain Institute, University of Queensland, Brisbane, QLD, Australia; ^9^Department of Psychiatry, Addenbrooke’s Hospital, University of Cambridge, Cambridge, United Kingdom; ^10^Department of Neurosurgery, Jaslok Hospital and Research Centre, Mumbai, India; ^11^Department of Neurology, Ruijin Hospital, Shanghai Jiao Tong University School of Medicine, Shanghai, China

**Keywords:** deep brain stimulation, Asia, Oceania, China, Japan, India

## Abstract

**Introduction**: To evaluate the current utilization and challenges in fully implementing the use of deep brain stimulation (DBS) treatment in Asia and Oceania.

**Methods**: We conducted a medical literature search to identify DBS research performed by investigators with a primary affiliation in Asian and Oceania countries between March 1, 2013, and March 1, 2019, followed by an international survey-based study. Additionally, we obtained added information regarding the DBS challenges and opportunities from the technology/industry perspective within China and Japan. We also described the current situation of DBS in India.

**Results**: Most publications (390/494; 78.95%) in the English language originated from East Asia. In West Asia, Turkey, Israel, and Iran accounted for most DBS publications. We found no publications from the remaining 35 Asian countries. Lack of community referrals to tertiary centers was identified as the most common limitation for the widespread use of DBS in Asia (68.97%). In China, despite an increasing number of centers performing DBS surgeries, most of them accomplished less than 10 cases per year. In contrast, the number of DBS cases in Japan has been decreasing. Centers offering DBS surgeries as well as corresponding fellowship training in India are limited.

**Conclusion**: Appropriate referrals, access, infrastructure, and the presence of full multidisciplinary DBS teams are common limitations of DBS in Asia. Most centers in China, Japan, and India performed less than 10 cases per year and a future study is expected to address the impact on quality in centers performing such few cases.

## Introduction

Deep brain stimulation (DBS) is a safe and effective treatment for medically refractory brain disorders. DBS was approved by the United States Food and Drug Administration as a treatment for essential tremor and Parkinson’s disease (PD) in 1997, dystonia in 2003, obsessive-compulsive disorder (OCD) in 2009, and epilepsy in 2018 (Okun, 2014; Li and Cook, 2018). DBS has been recently studied in small clinical trials as a potential treatment for other psychiatric disorders, e.g., major depression (Crowell et al., 2019), addiction (Chen et al., 2019; Zhang et al., 2019), and Tourette syndrome (Johnson et al., 2019; Ramirez-Zamora et al., 2019).

Expertise gained from surgical ablation has strongly influenced the clinical use of DBS, in particular the choice of targeted brain regions. The well-established long-term efficacy of bilateral high-frequency stimulation applied to different brain regions, coupled with the partially reversible nature of DBS and the possibility of reducing dopaminergic medications in PD patients has led to the global evolution toward DBS over ablative procedures to treat motor fluctuations and complications in PD (Krack et al., 2019). However, even in PD, less than 2% of eligible patients worldwide undergo DBS (Chan et al., 2014; Willis et al., 2014; Kestenbaum et al., 2015; Lee, 2015; Poortvliet et al., 2015; Ezat et al., 2017; Wagle Shukla et al., 2018).

Barriers to widespread adoption of DBS therapy include difficulties in predicting response and identifying appropriate candidates. There has been a reluctance of clinicians to refer patients for surgery, a shortage of personnel trained in DBS programming and issues about access to expert centers. Logistical barriers include expensive procedural costs and lack of insurance coverage. In many centers, long waiting lists for DBS surgery and the fear of brain surgery are common concerns, particularly in developing countries (Abosch et al., 2013).

A critical aspect of the effectiveness of DBS is patient selection. Another important aspect related to DBS success is choosing the appropriate brain target and surgical approach including factors such as the patient’s symptom profile, age, and cognitive status (Okun et al., 2004; Moro et al., 2016). These choices rely heavily on the expertise of the multidisciplinary team and vary from center to center. Most DBS studies have been published in European and North American countries (Hu et al., 2017) and there has been limited information regarding the local use and challenges of DBS in other world regions. More than two-thirds of people in the world reside in Asia and Oceania. To facilitate ongoing communication among these countries, the First 2019 DBS Think Tank East meeting was held on June 3rd, 2019 in Kyoto, Japan. A goal of this meeting was to provide a detailed survey of DBS practices within Asia and Oceania.

The objective of the international survey was to collect and share information on the current status of DBS surgery in Asia and Oceania and to achieve a better understanding of the local DBS challenges and opportunities. A complete literature review of DBS cases in Asia and Oceania was performed in addition to the survey. We present the results of the survey and the literature review.

## Materials and Methods

### Study Design

We designed this international survey study in March 2018 for the 2019 DBS Think Tank East meeting, which was held on June 3rd, 2019 in Kyoto, Japan. A 54-question internet-based survey was developed (designed with SurveyMonkey^1^) to ascertain various aspects of DBS surgical practice in Asian and Oceania regions including but not limited to: demographic information, DBS center information, number of different kinds of DBS surgeries (in 2018), team composition, specific surgical information, side effects, post-surgery management, number of patients, number of surgeries, as well as information on cost. The study conformed to the American Association for Public Opinion Research (AAPOR) regarding informed consent from the participants and compliance with human research ethics.

### Survey Question Formulation and Survey Tool

We initially conducted a literature review using the PubMed database searching for DBS studies published in English from Asia in the last 5 years at the stage of meeting preparation (i.e., between March 1, 2013, and March 1, 2018). The period was subsequently extended to March 1, 2019, to include more information before the meeting in June 2019. The Mesh term (or team) “DBS” was entered in the “Title/Abstract” field and country name, including “China,” “Japan,” “Korea,” “Mongolia,” etc. in the “Affiliation” field. Full country lists are included in Table 1. Basic science animal experiments, reviews, and publications using a language other than English were excluded. Searching results were manually verified. Only the corresponding author’s affiliation was used to define the “nationality” of the article. Subsequently, we contacted all corresponding authors by email to participate in this survey and to complete the electronic questionnaire. As stated in the invitation email, completion of the survey by participants was considered as implied consent. Responses were collected from May 17th, 2019 to July 8th, 2019.

**Table 1:** Publications and deep brain stimulation centers in Asia and Oceania.

No.	Country	No. of publications^a^	Publications^a^ per 10 million person	No. of centers identified from publications	Centers per 10 million person	Population (10 million)^b^	Ratio publications/ centers	GDP per capita (US dollars)^c^
1	China	210	1.46	23	0.16	143.38	9.13	9,770.85
2	Japan	91	7.17	22	1.73	12.69	4.14	39,286.74
3	Korea ^d^	89	17.37	10	1.95	5.12	8.90	31,362.75
4	Turkey	24	2.88	13	1.56	8.34	1.85	9,311.37
5	Australia	22	8.73	18	7.14	2.52	1.22	57,305.30
6	Iran	19	2.29	10	1.21	8.29	1.90	5,627.75
7	India	16	0.12	6	0.04	136.64	2.67	2,015.59
8	Israel	13	15.29	4	4.71	0.85	3.25	41,614.00
9	New Zealand	1	2.08	2	4.17	0.48	0.50	41,966.01
10	Saudi Arabia	3	0.87	2	0.58	3.43	1.50	23,219.13
11	Singapore	2	3.45	1	1.72	0.58	2.00	64,581.94
12	Philippines	2	0.19	1	0.09	10.81	2.00	3,102.71
13	Malaysia	1	0.31	1	0.31	3.19	1.00	1,1238.96
14	Nepal	1	0.35	1	0.35	2.86	1.00	1,025.80
15	Lebanon	0	0	0	0	0.69	NA	8,269.79
16	Thailand	0	0	0	0	6.96	NA	7,273.56
17	Afghanistan	0	0	0	0	3.80	NA	520.90
18	Armenia	0	0	0	0	0.30	NA	4,212.07
19	Azerbaijan	0	0	0	0	1.00	NA	4,721.18
20	Bahrain	0	0	0	0	0.16	NA	24,050.76
21	Bangladesh	0	0	0	0	16.30	NA	1,698.26
22	Bhutan	0	0	0	0	0.08	NA	3,360.27
23	Brunei	0	0	0	0	0.04	NA	31,627.74
24	Burma	0	0	0	0	5.40	NA	1,325.95
25	Cambodia	0	0	0	0	1.65	NA	1,512.13
26	Cyprus	0	0	0	0	0.12	NA	28,159.30
27	East Timor	0	0	0	0	0.13	NA	2,035.53
28	Georgia	0	0	0	0	0.40	NA	4,344.63
29	Indonesia	0	0	0	0	27.06	NA	3,893.60
30	Iraq	0	0	0	0	3.93	NA	5,878.04
31	Jordan	0	0	0	0	1.01	NA	4,247.77
32	Kazakhstan	0	0	0	0	1.86	NA	9,331.05
33	Kuwait	0	0	0	0	0.42	NA	34,243.95
34	Kyrgyzstan	0	0	0	0	0.64	NA	1,281.36
35	Laos	0	0	0	0	0.72	NA	2,567.54
36	Maldives	0	0	0	0	0.05	NA	10,223.64
37	Mongolia	0	0	0	0	0.32	NA	4,103.70
38	Oman	0	0	0	0	0.50	NA	16,418.93
39	Pakistan	0	0	0	0	21.66	NA	1,472.89
40	Palestine	0	0	0	0	0.50	NA	NA
41	Qatar/Katar	0	0	0	0	0.28	NA	69,026.47
42	Sri Lanka	0	0	0	0	2.13	NA	4,102.48
43	Syria	0	0	0	0	1.71	NA	2,032.62
44	Tajikistan	0	0	0	0	0.93	NA	826.62
45	The United Arab Emirates	0	0	0	0	0.98	NA	43,004.95
46	Turkmenistan	0	0	0	0	0.59	NA	6,966.64
47	Uzbekistan	0	0	0	0	3.30	NA	1,532.37
48	Vietnam	0	0	0	0	9.65	NA	2,563.82
49	Yemen	0	0	0	0	2.92	NA	944.41

*^a^We searched in Pubmed Database and included hurman studies published in English from Asia Between March 1, 2013, and March 1, 2019. The term “Deep brain stimulation” was entered in the “Title/Abstract” field and country name, including “China,” “Japan,” “Korea,” etc. in the “Affiliation” field. Basic science animal experiments, reviews, and publications using a language other than English were excluded. Supplementary information from manufacturers was also used for DBS center identification. ^b^Population data is obtained from World Population Prospects 2019 (total population, both sexes combined, as of 1 July 2019), United Nation (https://population.un.org/wpp/Download/Standard/Population/). ^c^Data is obtained from The World Bank, GDP *per capita* (most recent year https://data.worldbank.org/indicator/NY.GDP.PCAP.CD). ^d^“Korea” was used for both South Korea and North Korea. There were no publications from North Korea. Data on the population as well as GDP *per capita* include only South Korea*.

### Statistical Analysis

The survey results have been summarized descriptively. Quantitative data are presented as the median and interquartile range (IQR; 25th–75th percentile) and qualitative data are presented as “percentage.”

## Results

### DBS Development in Asia and Australia

We identified 494 studies, the majority of which [390/494 (78.95%)] were from East Asia (e.g., China, Japan, South Korea). Of the 114 centers identified from publications, 73 were in East Asia or Australia. For West Asia, Turkey, Israel, and Iran contributed to the most publications. There were thirty-five countries (71%) without any English publications on DBS according to our searching strategy (Table 1).

### Experience of Neurologists and Neurosurgeons

We received 37 answers from 32 DBS centers across nine countries. Most responses were from East Asia and Australia [27/37 (73%); Supplementary Table S1]. Most centers interviewed [31/36 (86%)] were major referral centers in their respective countries. The median number of DBS surgery centers interviewed was 14 (IQR 10–18) years (Supplementary Figure S1A).

Among the 37 respondents, 22 (60%) were neurosurgeons and 11 (30%) were neurologists. Additionally, two physiologists, one psychiatrist, and one neuro-engineer participated (Supplementary Figure S1C). Approximately half [18/37 (49%)] of respondents specialized in functional neurosurgery and approximately one-quarter [9/37 (24%)] identified as specialists in movement disorders; the rest practiced in the fields of neurophysiology, neuropsychiatry, and in general medicine (Supplementary Figure S1D). The median years of clinical experience was 13.0 (IQR 8.5–17.0) years, which was similar to the time engaged in utilizing neuromodulation techniques [14.0 (IQR 8.5–19.5) years; Supplementary Figure S1B]. Despite the relatively long history of DBS, 10/24 (42%) respondents had not performed more than 25 surgeries in 2018, and only one team reported performing more than 200 DBS surgeries in the previous year (Supplementary Figure S1E).

### Management of DBS: Surgical Team Members, Software and Hardware

Perioperative management of DBS is usually a multidisciplinary effort that involves neurologists, neurosurgeons, physical therapists, psychiatrists, psychologists, and case managers. Our data suggested that in Asia the team usually involves more neurologists [3 (IQR 2–3.75)] than neurosurgeons [2 (IQR 1–3); Supplementary Table S2, Supplementary Figure S2A]. The number of physicians or therapists increased at centers with larger surgical volumes (Supplementary Table S3).

Most centers [31/33 (94%)] used microelectrode recording (MER) to identify appropriate brain areas for implantation. Only one center preferred to implant using intraoperative magnetic resonance imaging (MRI) or computed tomography (CT) for lead localization (Supplementary Figure S2B). Leksell SurgiPlan and Stealth were the two most popular planning software programs utilized for stereotactic neurosurgery [26/32 (81%); Supplementary Figure S2C]. Approximately 5% (95% CI 5–10%) of all procedures were reported as unsatisfactory related to hardware (1.6% of all cases, 95% CI 1–2%) or surgery-related (1% of all cases, 95% CI 1–2%) complications (Supplementary Figure S2D). Neurologists were responsible for postoperative DBS programming in over half of the centers (21/32 [66%]). Most teams offered DBS programming, DBS troubleshooting, and non-DBS outpatient movement disorder evaluations [28/29 (97%), 22/29 (76%), and 23/29 (79%), respectively]. A large proportion of centers also endorsed conducting preclinical research [23/29 (80%)]. Nearly half of the DBS centers [14/29 (48%)] did not provide rehabilitation services for DBS patients. It was unclear if the teams worked consistently in a multidisciplinary fashion and how multidisciplinarity was defined.

Since DBS is a well-established treatment option for PD, essential tremor (ET), and dystonia, we further investigated and compared the preferred clinical workflow for these three disorders. Simultaneous bilateral lead implantation was predominantly performed for PD [28/29 (97%)], ET [23/29 (79%)], and dystonia [28/29 (97%); Supplementary Figure S2E].

Various DBS devices are available for clinicians and patients to use. Device reliability was the most important factor in the decision [23/29 (80%)], followed by customer support [17/29 (59%)] and programming feasibility [16/19 (55%)]. However, patient preference, as well as insurance coverage (i.e., the final cost to the patient and other funding considerations), played a minor role. Unsurprisingly, economic issues affected DBS centers in developing countries (*p* = 0.000023). Only one center in Iran and one in Turkey decided on device selection based on government-related factors (Supplementary Table S4). Insurance and government support covered the expenses for device and hospital services in all of the DBS centers in developed countries (*p* = 0.005) making DBS potentially more accessible to patients in these regions (Supplementary Table S5). Conversely, in developing countries, out-of-pocket payment served as the main form of reimbursement (*p* = 0.000023).

### Major Obstacles for DBS Accessibility and Development

More than half of responders [16/29 (55%)] indicated that the main limiting factors for access to surgery included: (1) insufficient referrals due to limited understanding of DBS by general medical providers [20/29 (69%)]; (2) fear of brain surgery by patients [17/29 (59%)]; and (3) high cost of the device and procedure [12/29 (41%)], with cost issues most notable in developing countries (*p* = 0.001; Supplementary Table S6). DBS is an established treatment for some neurological movement-related diseases and is a promising investigational approach for psychiatric disorders (e.g., OCD and depression). Lack of funding was a major barrier to increasing scientific research. The survey also uncovered difficulties in recruiting patients and in publishing results (Supplementary Table S7).

### DBS in China and Japan

**Figure 1:**
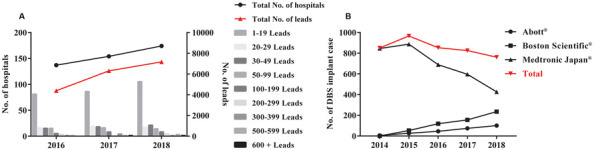
The number of deep brain stimulation (DBS) implant cases in China **(A)** and Japan **(B)**.** (A)** DBS surgery became more popular in China in recent years. Histogram with different gray bars shows the number of hospitals performing DBS electrode implantation each year on the left Y-axis. The total number of hospitals is then indicated by the black “•” The total number of implanted leads is shown by the red “•” on the right Y-Axis. **(B)** Japan performed less DBS surgeries since 2015. Red “▾” shows the total number of DBS implanted cases between 2014 and 2018 in Japan. Black “•,” “▪,” and “▴” indicate each number of DBS implant cases of three main manufacturers (Abott^®^, Boston Scientific^®^, and Medtronic Japan^®^, respectively) in Japan.

DBS was introduced in China in 1998 (source of information Beijing Tiantan Hospital) and has undergone considerable development in the past 20 years. In the last 3 years, the number of DBS centers in China has increased, with over 200 DBS leads implanted per year/center. More than 100 Chinese hospitals performed less than 10 cases annually (Figure 1A). In contrast, the number of DBS implantations in Japan has decreased since 2015 (Figure 1B). While 966 cases underwent DBS implantation in 2015, there were only 761 cases in 2018 (according to Medtronic Japan). Among the 44 institutions approved as training centers (≥18 cases/3 years) by the Japanese Society for Stereotactic and Functional Neurosurgery (JSSFN) in 2019, most centers performed less than two cases per month. Although our survey did not investigate factors associated with these small numbers, possibly, these numbers were influenced by the introduction of focused ultrasound (FUS) and other advanced therapies, e.g., levodopa gastrointestinal gel. Since November 2016, when the FUS obtained approval for the treatment of essential tremor, 210 cases were treated with FUS (November 2016 to October 2019).

### DBS in India

The first DBS surgery in India was performed in a private hospital, Jaslok Hospital and Research Center, by Prof. Paresh Doshi (Ganapathy, 2013). There has been a steady increase in interest in DBS over the past 20 years. Currently, 3/4 premier state-run neuroscience centers perform DBS surgeries regularly, which include All India Institute of Medical Sciences in New Delhi, National Institute of Mental and Neurosciences (NIMHANS) in Bengaluru, and Sree Chitra Tirunal Institute for Medical Sciences and Technology in Trivandrum. In total, 12 centers offer DBS with at least 10 DBS surgeries occurring per year, and another 12–15 centers with much fewer numbers of cases per year. Generally, most provide DBS surgeries for all movement disorders. Two centers, Jaslok Hospital and NIMHANS, also perform DBS for psychiatric disorders. The DBS surgical programs in most hospitals are actively supported by movement disorder specialists. Though not all surgeons have formal fellowship training in functional neurosurgery, the neurologists in most centers have obtained fellowship training specialized in movement disorders, either in India or overseas. The movement disorder fellowship is offered in 8–10 centers in India, whereas Jaslok Hospital provides the unique functional neurosurgical training. Three societies, the Indian Society for Stereotactic and Functional Neurosurgery, the Neuromodulation Society, and the Movement Disorders Society of India, are involved in DBS in India. Two major companies, Medtronic and Boston Scientific, provide most of the DBS implants in India. SceneRay and Abbott are, however, just entering the market. The use of DBS has increased and though an exact estimate cannot be made, approximately 450 DBS surgeries/year are being performed in India.

India has a legacy of lesional surgeries for movement disorders (Doshi, 2009). Most centers perform such interventions mainly for unilateral dystonia, including task-dystonia, and tremors (Doshi et al., 2017). All centers refrain from offering bilateral lesional surgeries. Compared to developed nations, the number of patients undergoing lesional surgery (or DBS) for essential tremors is very low.

Most DBS surgeries are self-funded. However, there is currently an increasing tendency for insurance reimbursements. Owing to the social customs of family support in India, many PD patients are funded by their children who are now earning adequately. There is also a growing trend towards seeking DBS for a moderately advanced disease by neurologists and patients compared to 10 years ago.

## Discussion

These data provide a detailed and previously unknown description of DBS practices and practice patterns in centers from Asia and Oceania. These data will serve as a reference for addressing the challenges and limitations of DBS in these regions. We also hope that through the DBS Think Tank East there will be dialogue including relevant stakeholders and medical societies to discuss potential solutions and opportunities to the challenges uncovered by the survey. Though outcomes including clinical effectiveness and adverse events were not obtained, we believe that the information highlights important challenges and barriers in the field.

Of the 51 countries identified, only Japan, Korea, Israel, Australia, and New Zealand are considered “developed countries” (UNDP, 2018). These are also the most active countries in terms of DBS usage for both clinical practice and research. China has had a rapid increase in surgical volume and publications since 2009. This increase coincides with the period when local manufacturers, including PINS and SeneRay, produced less expensive and more accessible products. A DBS neurostimulator (battery source) and bilateral leads have an estimated expense of 250,000–350,000 Renminbi (RMB; approximately $32,000–42,000 USD). It is estimated that from 1998 to 2003, approximately 312 patients in China received DBS; from 2003 to 2009, this number increased to approximately 1,700. Following the commercialization of products from Chinese enterprises, over 15,000 patients have received DBS, and over 180 hospitals now offer DBS. Finally, an increasing number of institutions have launched DBS programs in China, but most centers perform less than 10 cases per year and are unclear whether proper teams and adequate quality can be maintained at hospitals with such low volumes. In contrast, the number of DBS cases in Japan has been decreasing over the last few years. This might be due in part to the advent of FUS, but more data are required to understand the underpinnings of the recent decline.

Research on DBS in Asia has been limited. The major barrier for DBS research has been a lack of funding from governments and industry. Our data suggest that 77% (38) of Asian countries have not published on DBS. In comparison, 402 DBS publications from Canada alone were revealed when employing a similar search strategy to ours. DBS in Asia is evolving and many barriers and challenges are remaining. Many countries still do not have access to DBS and most DBS publications favor East Asia and Oceania. In West Asia, Turkey, Israel, and Iran accounted for most of the DBS publications.

Financial cost continues to be a significant barrier to DBS research and its widespread clinical adoption. The hardware is expensive, the surgery is costly, and when factoring in follow-up care these factors add up to a significant expense. Some experts have however argued that long-term there can be savings made from DBS (e.g., fewer medications, decreased morbidity). Cost is problematic in the developing world, where few citizens can afford the hardware and treatment costs. This situation is unfortunate given the high prevalence of movement disorders and other conditions amenable to DBS intervention. The development of low-cost hardware may aid in ameliorating this problem. Lower cost hardware, is, according to our data, mostly available in China.

One issue that stood out was the lack of referrals (20/29; 69%) for DBS. This was a major impediment to DBS clinical practice in these regions. Once referred and implanted another interesting issue that emerged was that DBS programming was performed by a mix of 66% neurologists and 34% neurosurgeons. This pattern of care differs from practice patterns in Western countries, where teams are composed mainly of nurses and neurologists and it is rare to have neurosurgeons programming patients. It was surprising that many Asian centers did not have full multidisciplinary teams. The evolution of DBS in these regions may eventually favor larger expert centers; however, this will be addressed by a future survey. Educational outreach is a critical unmet need across these regions and is necessary to improve access and understanding of DBS therapies.

Our survey has several limitations. First, we are only able to obtain information from limited countries. We attempted to gather information about manufacturers used in Asian countries. However, this information is considered confidential by some manufacturers. Similarly, the number of patients implanted would be a better indicator of the popularization of DBS, but some manufacturers prefer to share with us the number of leads. Second, we selected the dates in our search strategy based on likely capturing DBS centers with “active” and publications in recent years as supposed to include all articles. We are aware that this strategy may filter out some centers with prior DBS experience and publications before 2013 (e.g., paper from Thailand published in 2010; Nunta-Aree et al., 2010).

In clinical practice, microelectrode recordings remain widely used for target refinement during surgery (94%) and the DBS programming team consists primarily of neurologists (66%) and neurosurgeons (34%). There was a lack of community referrals to tertiary centers and this factor was identified as the most common limitation for the widespread use of DBS in Asia. In China, there has been an increasing number of centers performing DBS surgeries. In contrast, the number of DBS cases in Japan has been decreasing, which might be related to the use of novel non-craniotomy approach FUS lesioning and the availability of other advanced treatment options. Whether this new technology has impacted DBS use was not directly examined by our survey. Appropriate referrals, access, infrastructure, and the lack of full multidisciplinary DBS teams are common limitations for DBS in Asia. Most centers in China and Japan performed less than 10 DBS cases per year and it is unclear if low volumes will result in suboptimal outcomes, which is a worry of experts in the field. We know that the success of DBS relies on excellent patient selection and the utilization of large multidisciplinary teams. Such teams seem to be lacking in Asia and their establishment this will be a critical next step for the field to evolve in this region. Global educational and training programs will be needed to scale up DBS and a focused effort will be necessary to address financial barriers and improve the quality of care delivery.

## Data Availability Statement

The datasets generated for this study are available on request to the corresponding author.

## Ethics Statement

The studies involving humans were reviewed and approved by the ethics committee of Ruijin Hospital Shanghai Jiao Tong University School of Medicine. Completion of the survey by participants was considered as implied consent, which adhered to the guidelines for the American Association for Public Opinion Research (AAPOR).

## Author Contributions

CZ, AR-Z, BS, and MO: conception and design. CZ, ZL, and YL: analysis and interpretation. YL, DL, FM, JC, TM, TI, SF, GO, TC, VV, PD, YW, JL, JZ, KF, BP, LA, and AW: data collection. CZ, ZL, AR-Z, and MO: writing the article. AR-Z, BS, and MO: critical revision of the article. ZL and YL: statistical analysis. All authors have read and approved the final version of the article.

## Conflict of Interest

The authors declare that the research was conducted in the absence of any commercial or financial relationships that could be construed as a potential conflict of interest.

## Footnotes

^1^https://www.surveymonkey.com/r/eastdbs
