# Supplementary material for: An International Survey of Deep Brain Stimulation Utilization in Asia and Oceania: The DBS Think Tank East
Source: Front Hum Neurosci. 2020 Jul 6;14:162. doi: 10.3389/fnhum.2020.00162 (PMC7357800; doi:10.3389/fnhum.2020.00162)
Supplement: Supplementary file 1 [file Table_1.DOCX]

**
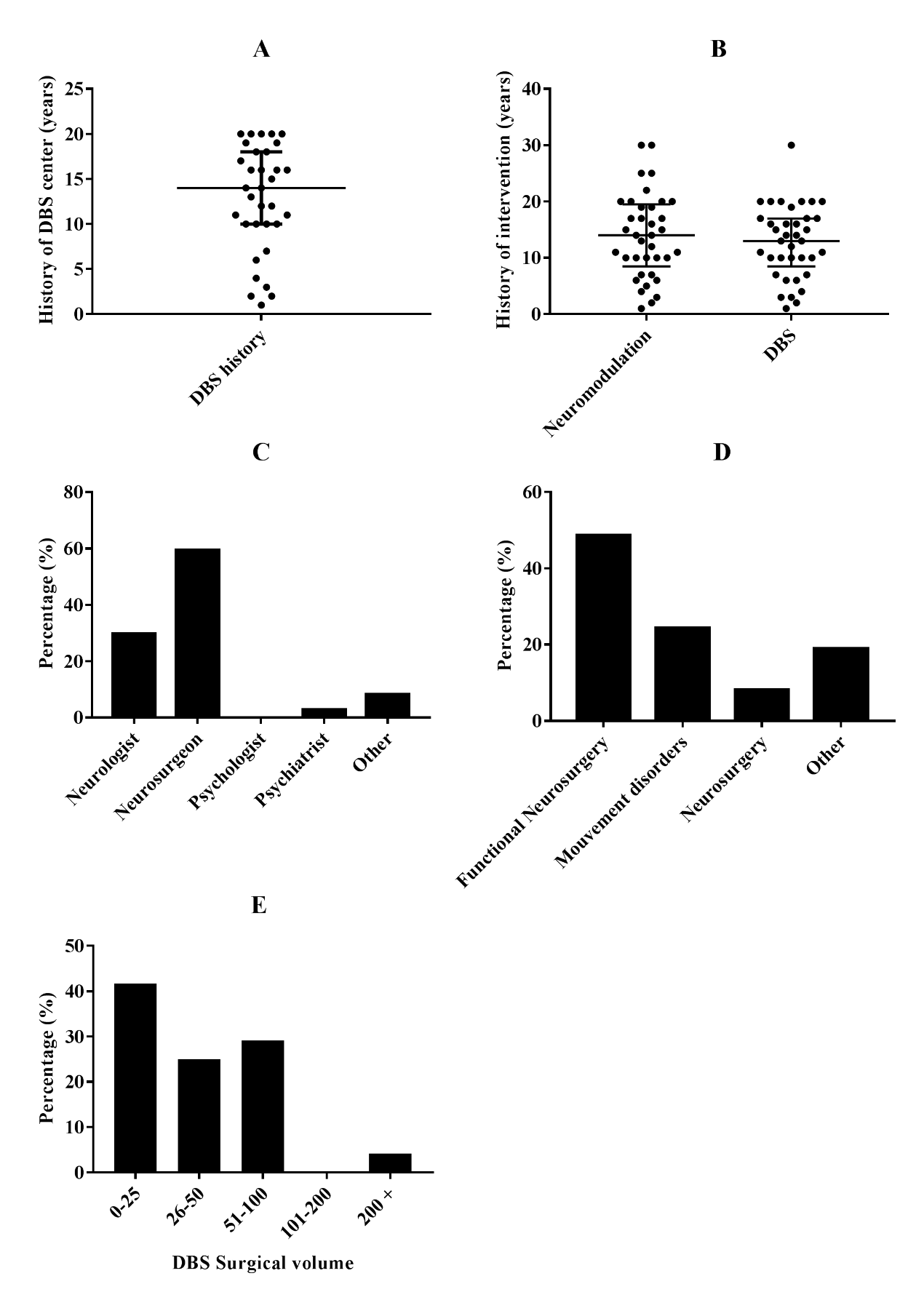
**

**Supplementary Figure 1. Characteristics of respondents and interviewed DBS surgery centers.** (A) - (B): History of deep brain stimulation (DBS) centers (A) and history of interventions performed by respondents (B). The black dots indicate individual data. The median line and the lower and upper border lines indicate the median value and the 25^th^ and 75^th^ percentiles, respectively. (C) – (D): Occupation (C) and specialty (D) of the thirty-seven respondents. (E): Surgical volumes accomplished by interviewed DBS surgery centers^a^ in 2018.

^a^: Thirty-six respondents answered this questions and one skipped.

**
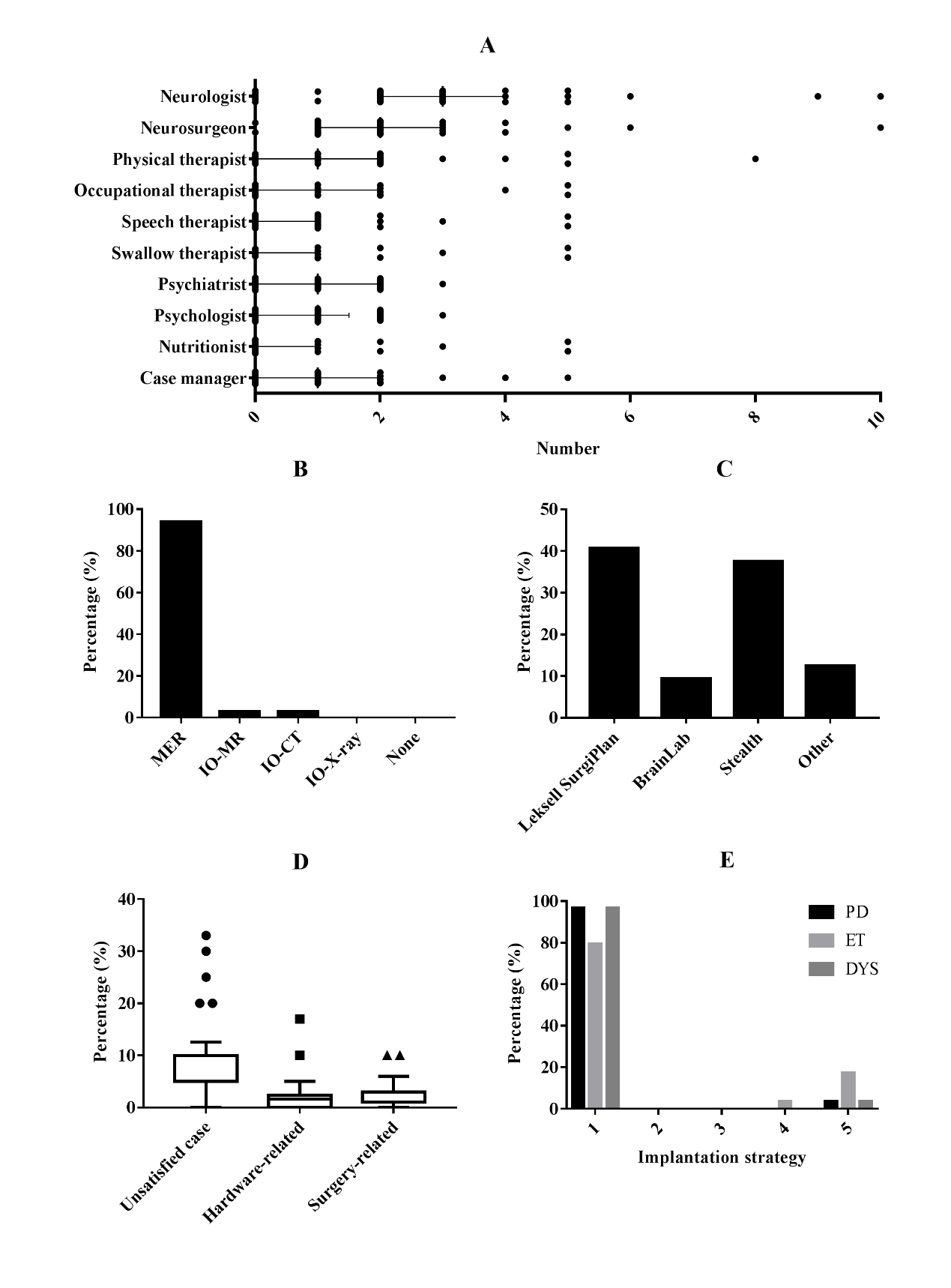
Supplementary Figure 2. Management of DBS: from surgical team composition to software and hardware equipment.** (A): Composition of a deep brain stimulation-specialized surgery team. The black dots indicate the number of each type of specialist in interviewed teams. The median line and the lower and upper border lines indicate the median value and the 25^th^ and 75^th^ percentiles, respectively. (B): Intra-operative modalities for identification of brain areas of interest in interviewed DBS surgery centers^a^. (C): Planning software preference in interviewed deep brain stimulation surgery centers^b^. (D): Complication rate related to hardware and surgery procedures^c^. The lower and upper borders of the boxes indicate the 25^th^ and 75^th^ percentiles, respectively. The median value is shown by the line inside the box. For “Unsatisfied outcome” and “Surgery-related”, the median value overlapped with the value of 25^th^ percentile. The black circles, squares, and triangles indicate outlier values for “Unsatisfied outcome”, “Hardware-related”, and “Surgery-related”, respectively. (E): Deep brain stimulation electrode implantation strategy for Parkinson’s disease (PD), essential tremor (ET), and dystonia (DYS)^d^. 1: Bilateral simultaneous; 2: bilateral, staged (weekly); 3: bilateral, staged (monthly); 4: bilateral, with long staging (intervals of at least 6 months); 5: unilateral, with patient reassessment before contralateral side implantation.

^a^: Thirty-three respondents answered this questions and four skipped.

^b^: Thirty-two respondents answered this questions and five skipped.

^c^: Thirty respondents answered this questions and seven skipped.

^d^: Twenty-nine respondents answered this question and eight skipped.
