# Supplementary material for: An International Survey of Deep Brain Stimulation Utilization in Asia and Oceania: The DBS Think Tank East
Source: Front Hum Neurosci. 2020 Jul 6;14:162. doi: 10.3389/fnhum.2020.00162 (PMC7357800; doi:10.3389/fnhum.2020.00162)
Supplement: Supplementary file 2 [file Table_2.DOCX]

**Supplementary Table 1. Distribution of Interviewees by Country.**

| **Country** | **No. of Responses (%)** |
| --- | --- |
| Japan | 11 (30%) |
| Korea | 8 (22%) |
| Australia | 4 (11%) |
| China | 4 (11%) |
| India | 5 (14%) |
| Turkey | 2 (5%) |
| Philippines | 1 (3%) |
| Israel | 1 (3%) |
| Iran | 1 (3%) |
| Total | 37 (100%) |

**Supplementary Table 2. Descriptive Statistics of Team Members per Deep Brain Stimulation Surgery Team^a^.**

| **Team Member** | **Total No.** | **Median No. (Interquartile Range)** |
| --- | --- | --- |
| **Neurologist** | 99 | 3 (2–3.75) |
| **Neurosurgeon** | 78 | 2 (1–3) |
| **Physical therapist** | 45 | 1 (0–2) |
| **Occupational therapist** | 32 | 0 (0–1.75) |
| **Speech therapist** | 29 | 0 (0–1) |
| **Swallow therapist** | 23 | 0 (0–1) |
| **Psychiatrist** | 32 | 1 (0–2) |
| **Psychologist** | 29 | 1 (0–1) |
| **Nutritionist** | 23 | 0 (0–1) |
| **Case manager** | 33 | 1 (0–1.75) |

^a^Total No. of answered teams: 34; No. of skipped teams: 3

**Supplementary Table 3. Deep Brain Stimulation Team Composition by Surgical Volume.**

|  | **Team Members** | | | | | | | | | |
| --- | --- | --- | --- | --- | --- | --- | --- | --- | --- | --- |
| **No. of Surgeries** | **Neurologist** | **Neurosurgeon** | **Physical Therapist** | **Occupational Therapist** | **Speech Therapist** | **Swallow Therapist** | **Psychiatrist** | **Psychologist** | **Nutritionist** | **Case Manager** |
| **0–25** | 3.2 | 2.2 | 0.8 | 0.6 | 0.3 | 0.2 | 1.0 | 0.9 | 0.1 | 0.3 |
| **26–50** | 2.8 | 1.7 | 1.3 | 1.0 | 0.7 | 0.5 | 1.3 | 1.3 | 0.7 | 1.3 |
| **5–100** | 1.9 | 1.9 | 2.0 | 1.8 | 1.7 | 1.5 | 1.0 | 0.9 | 1.3 | 0.7 |
| **200+** | 5.0 | 10.0 | 0 | 0 | 0 | 0 | 0 | 1.0 | 0 | 5.0 |

**Supplementary Table 4. Deciding Factors for Choosing the Implanted Deep Brain Stimulation Devices by Respondents.**

| **Answer choices** | **Response** | **Count** | | | ***p*-value ^a^** |
| --- | --- | --- | --- | --- | --- |
|  |  | **Total** | **Developed** | **Developing** |  |
| 1. Patient preference | 24.14% | 7 | 5 | 2 | 1.000 |
| 2. Customer support (company representatives readily available for implantation and patient care) | 58.62% | 17 | 10 | 7 | 0.234 |
| 3. Device reliability (hardware malfunctioning rates, rates of fractured leads, etc.) | 79.31% | 23 | 16 | 7 | 1.000 |
| 4. Ease of programming as an outpatient | 55.17% | 16 | 10 | 6 | 0.454 |
| 5. Final cost for the patient | 20.69% | 6 | 0 | 6 | 0.000177 |
| 6. Research funding from company | 3.45% | 1 | 0 | 1 | 0.310 |
| 7. Supply availability at your institution (whichever devices they have available in stock at the time at your hospital) | 20.69% | 6 | 4 | 2 | 1.000 |
| 8. Government determined (city, state, or country-based contracts with a company for ordering certain number of devices from a specific company) | 6.90% | 2 | 0 | 2 | 0.089 |
|  |  |  |  |  |  |
|  | **Answered** | 29 | 20 | 9 |  |
|  | **Skipped** | 8 | 4 | 4 |  |

^a^: *p*-value indicates the difference of response between DBS centers from developed and developing countries. If we combine the answer choice No.5 and No.6 as economic factors, the *p*-value between developed and developing countries account for 0.000023.

**Supplementary Table 5. Main Forms of Reimbursement for Deep Brain Stimulation Surgery**

| **Answer choices** | **Response** | **Count** | | | ***p*-value ^a^** |
| --- | --- | --- | --- | --- | --- |
|  |  | **Total** | **Developed** | **Developing** |  |
| 1. Out-of-pocket payment for the entire procedure and care | 24.14% | 7 | 0 | 7 | 0.000023 |
| 2. Government-sponsored program with minimal to no cost to patients | 37.93% | 11 | 10 | 1 | 0.096 |
| 3. Insurance-based practice (insurance coverage of the entire procedure and patient care, with minimal to no cost for patients) | 48.28% | 14 | 10 | 4 | 1.000 |
| 4. Military program/hospital coverage | 0.00% | 0 | 0 | 0 | NA |
| 5. Mix (insurance or government used for hospital services and device coverage) and physician pay as an out-of-pocket cost to patients | 17.24% | 5 | 3 | 2 | 0.633 |
| 6. Other (please specify) | 0.00% | 0 | 0 | 0 | NA |
|  | **Answered** | 29 | 20 | 9 |  |
|  | **Skipped** | 8 | 4 | 4 |  |

^a^: *p*-value indicates the difference of response between DBS centers from developed and developing countries. If we combine the answer choice No.2, No.3, and No.5 as coverage availability, the *p*-value between developed and developing countries account for 0.005.

**Supplementary Table 6. Main Impediments for the Use of Deep Brain Stimulation in Asia and Australia.**

| **Answer choices** | **Response** | **Count** | | | ***p*-value ^a^** |
| --- | --- | --- | --- | --- | --- |
|  |  | **Total** | **Developed** | **Developing** |  |
| 1. DBS is not regulated/approved in my country | 3.45% | 1 | 0 | 1 | 0.31 |
| 2. Cost | 41.38% | 12 | 4 | 8 | 0.001 |
| 3. Not receiving appropriate referrals from other providers / lack of medical community education | 68.97% | 20 | 14 | 6 | 1.000 |
| 4. Patient education / fear of brain surgery | 58.62% | 17 | 12 | 5 | 1.000 |
| 5. Lack of appropriate outpatient follow-up (e.g. patients may get the device, but return to a different state, or remote area, and there is nobody to program or provide further care) | 6.90% | 2 | 1 | 1 | 0.532 |
| 6. Other | 6.90% | 2 | 1 | 1 | NA |
|  | **Answered** | 29 | 20 | 9 |  |
|  | **Skipped** | 8 | 4 | 4 |  |

^a^: *p*-value indicates the difference of response between DBS centers from developed and developing countries.

**Supplementary Table 7. Barriers for Research as Claimed by Respondents.**

| **Answer choices** | **Response** | **Count** | | | ***p*-value ^a^** |
| --- | --- | --- | --- | --- | --- |
|  |  | **Total** | **Developed** | **Developing** |  |
| 1. Lack of government funding | 58.62% | 17 | 13 | 4 | 0.422 |
| 2. Lack of industry support | 44.83% | 13 | 8 | 5 | 0.668 |
| 3. Difficulties in recruiting patients | 37.93% | 11 | 8 | 3 | 1.000 |
| 4. We can start projects, but lack of support makes us interrupt them before finishing | 10.34% | 3 | 2 | 1 | 1.000 |
| 5. We find good results, but have difficulties publishing | 27.59% | 8 | 5 | 3 | 0.675 |
| 6. We have mostly case reports and series, and those are difficult to be accepted by journals | 27.59% | 8 | 5 | 3 | 0.675 |
| 7. We have no difficulties with publication | 3.45% | 1 | 1 | 0 | 1.000 |
| 8. We do not perform research | 0.00% | 0 | 0 | 0 | NA |
| 9. Other | 3.45% | 1 | 1 | 0 | NA |
|  | **Answered** | 29 | 20 | 9 |  |
|  | **Skipped** | 8 | 4 | 4 |  |

^a^: *p*-value indicates the difference of response between DBS centers from developed and developing countries.
