# Supplementary material for: An International Survey of Deep Brain Stimulation Utilization in Asia and Oceania: The DBS Think Tank East
Source: Front Hum Neurosci. 2020 Jul 6;14:162. doi: 10.3389/fnhum.2020.00162 (PMC7357800; doi:10.3389/fnhum.2020.00162)
Supplement: Supplementary file 3 [file Table_3.DOCX]

Search strategy: we searched for DBS studies written in English from Asia between March 1, 2013 to March 1, 2019 in Pubmed database. The term ‘Deep brain stimulation’ was entered in the ‘Title/Abstract’ field and country name, including ‘China’, ‘Japan’, ‘Korea’, etc. in the ‘Affiliation’ field. Basic science animal experiments, reviews, and publications using language other than English were excluded.

Supplementary information from manufacturers was also used for DBS center identification.

Centers identified are listed as follows:

1. **East Asia**

| **China** |
| --- |
| 1. Anhui Provincial Hospital Affiliated to Anhui Medical University, Hefei, China 2. Beijing Tian Tan Hospital, Beijing, China 3. China Medical University Hospital, Taichung, Taiwan 4. First Affiliated Hospital, Sun Yat-sen University, Guangzhou, China 5. General Hospital of Shenyang Military Area Command, Shenyang, China 6. Hebei General Hospital, Shijiazhuang, China 7. Huashan Hospital, Shanghai, China 8. Nanfang Hospital, Southern Medical University, Guangzhou, China 9. Peking Union Medical College Hospital, Chinese Academy of Medical Sciences, Beijing, China 10. PLA General Hospital, Beijing China 11. Prince of Wales Hospital, Hong Kong, China 12. Ruijin Hospital, Shanghai, China 13. Shenzhen Second People's Hospital, Shenzhen, China 14. Tangdu Hospital, Xi’an, China 15. The First Affiliated Hospital of Fujian Medical University, Fuzhou, China 16. Tainan Municipal An-Nan Hospital-China Medical University, Tainan, Taiwan 17. Tzu-Chi General Hospital, Taiwan, China 18. The Second Xiangya Hospital, Changsha, China 19. West China Hospital, Sichuan University, Chengdu, China 20. Xuanwu Hospital, Beijing, China 21. Yuquan Hospital, Tsinghua University, Beijing, China 22. ZhongNan Hospital WuHan University, WuHan, China 23. Zhujiang Hospital, Southern Medical University, Guangzhou, China |

| **Korea** |
| --- |
| 1. Asan Medical Center, Seoul, Korea 2. Busan Paik Hospital, Busan, Korea 3. Catholic Neuroscience Institute, Seoul, Republic of Korea 4. Guro Hospital, Seoul, Republic of Korea 5. Hallym University Sacred Heart Hospital, Anyang, Republic of Korea 6. Incheon St. Mary’s Hospital, Incheon, South Korea 7. Korea University College of Medicine, Seoul, Korea 8. Samsung Medical Center, Seoul, Republic of Korea 9. Seoul National University Hospital, Seoul, Korea 10. Brain Research Institute, Yonsei University College of Medicine, Seoul, Korea |

| **Japan** |
| --- |
| 1. Chiba University Graduate School of Medicine, Chiba, Japan 2. Fukui Red Cross Hospital, Fukui, Japan 3. Fukuoka University, Fukuoka, Japan 4. Hamamatsu University School of Medicine, Hamamatsu-city, Shizuoka, Japan 5. Juntendo University School of Medicine, Tokyo, Japan 6. Kinki University, School of Medicine 7. Kitano Hospital, Osaka, Japan 8. Kumamoto University Hospital, Kumamoto, Japan 9. Nagasaki Kawatana Medical Center, Nagasaki, Japan 10. Nagoya University Graduate School of Medicine, Japan 11. National Center Hospital, Tokyo, Japan 12. Nihon University School of Medicine, Tokyo , Japan 13. Osaka University Graduate School of Medicine, Osaka, Japan 14. Sakura Medical Center, Sakura, Japan 15. Sapporo Medical University Hospital, Sapporo, Japan 16. Saitama Medical University, Saitama, Japan 17. Keio University School of Medicine, Tokyo, Japan 18. Tokyo Metropolitan Neurological Hospital, Tokyo, Japan 19. Tokyo Women’s Medical University, Tokyo, Japan 20. Institute of Health Biosciences, University of Tokushima, Tokushima , Japan 21. Graduate School of Medicine and Pharmaceutical Science, University of Toyama, Toyama , Japan 22. US Naval Hospital, Okinawa, Japan |

1. **South Asia and Southeast Asia**

| **Philippines** |
| --- |
| 1. Philippine Movement Disorder Surgery Center (PhilMove) and Department of Medicine, Cardinal Santos Medical Center, San Juan City, Philippines |

| **Singapore** |
| --- |
| 1. Parkinson's Disease and Movement Disorders Center, National Neuroscience Institute, Singapore |

| **India** |
| --- |
| 1. Neurosciences Center, AIIMS, New Delhi, India 2. Comprehensive Care Centre for Movement Disorders, Sree Chitra Tirunal Institute for Medical Sciences and Technology, Thiruvananthapuram, Kerala, India 3. National Institute of Mental Health and Neurosciences (NIMHANS), Karnataka, India 4. Jaslok Hospital and Research Centre, India 5. Columbia Asia Hospital, Bangalore, India 6. Yashoda Hospitals, Secunderabad, Telangana, India |

| **Malaysia** |
| --- |
| 1. Department of Neurosciences, School of Medical Sciences, Universiti Sains Malaysia, 16150 Kubang Kerian, Kelantan, Malaysia |

| **Nepal** |
| --- |
| 1. Annapurna Neurological Institute and Allied Sciences, Kathmandu, Nepal |

1. **Middle Asia and West Asia**

| **Iran** |
| --- |
| 1. Kowsar Hospital, Shiraz, Iran 2. Shiraz University of Medical Sciences, Shiraz, Iran 3. Hazrat Rasool Hospital, Iran University of Medical Sciences, Tehran, Iran 4. Baqiyatallah University of Medical Sciences, Tehran, Iran 5. Rasoul-E-Akram Hospital, Teheran University of Medical Sciences, Tehran, Iran 6. Firoozgar Hospital, Iran University of Medical Sciences, Tehran, Iran 7. Qom University of Medical Science, Qom, Iran 8. Kermanshah University of Medical Sciences, Kermanshah, Iran 9. School of Behavioral Sciences and Mental Health, Iran University of Medical Sciences, Tehran, Iran 10. Chamran Hospital, Shiraz, Iran |

| **Turkey** |
| --- |
| 1. Group Florence Nightingale Hospitals, Istanbul, Turkey 2. Mustafa Kemal University, Anatakya, Hatay, Turkey. 3. Dokuz Eylül University, Izmir, Turkey 4. Liv Hospital, Istanbul, Turkey 5. Ondokuz Mayis University, Samsun, Turkey 6. Ege University Medical Faculty, Bornova-Izmir, Turkey 7. Selçuk University, Konya, Turkey 8. Gumussuyu Military Hospital, Istanbul, Turkey 9. Vezirköprü State Hospital, Samsun, Turkey 10. Ankara University School of Medicine, Ankara, Turkey 11. Marmara University Faculty of Medicine, Maltepe, Turkey 12. Pamukkale University, Denizli, Turkey 13. Istanbul Education and Research Hospital, Istanbul, Turkey |

| **Israel** |
| --- |
| 1. Tel Aviv Sourasky Medical Center, Tel Aviv, Israel 2. Hadassah Hebrew University Medical Center, Jerusalem, Israel 3. Multidisciplinary Brain Research Center, Bar-Ilan University, Ramat-Gan, Israel 4. Sheba Medical Center, Tel Hashomer, Israel |

| **Saudi Arabia** |
| --- |
| 1. Jazan University, Jazan, Saudi Arabia 2. Taibah University, Madinah Munawarah, Saudi Arabia |

1. **Australia and New Zealand**

| **Australia** |
| --- |
| 1. St Andrew's War Memorial Hospital 2. Prince of Wales Hospital 3. Women’s and Children’s Hospital 4. Mater Hospital 5. Asia Pacific Centre for Neuromodulation, Queensland Brain Institute 6. Princess Alexandra Hospital 7. Westmead Hospital 8. St. Vincent's Hospital 9. Alfred Hospital 10. Royal Adelaide Hospital 11. Royal Perth Hospital 12. Queen Elizabeth Hospital 13. St. Andrews and Wesley Hospitals 14. Gairdner Hospital 15. Royal Brisbane & Women's Hospital 16. Western Australian Neuroscience Research Institute 17. North Shore Medical Centre 18. Royal Melbourne Hospital |

| **New Zealand** |
| --- |
| 1. Auckland City Hospital, Auckland, New Zealand 2. Dunedin School of Medicine, University of Otago, New Zealand |
